# Selinexor in combination with pomalidomide and dexamethasone for the treatment of primary plasma cell leukemia with 1q21+ abnormality: A case report

**DOI:** 10.1097/MD.0000000000040447

**Published:** 2024-11-15

**Authors:** Wenxia Fan, Lei Wang, Xinyou Wang, Ying Liu, Mingling Sun, Nadia Abduklimu, Rui Zhang, Ming Jiang, Xinhong Guo

**Affiliations:** a Hematologic Disease Center, The First Affiliated Hospital of Xinjiang Medical University, Xinjiang Uygur Autonomous Region Research Institute of Hematology, Urumqi, China.

**Keywords:** 1q21+ abnormality, dexamethasone, high-risk genetic abnormalities, pomalidomide, primary plasma cell leukemia, selinexor

## Abstract

**Rationale:**

Primary plasma cell leukemia is a rare and highly aggressive malignancy of the blood system, with rapid disease progression and a high early mortality rate. Currently, there is no recognized therapeutic regimen, leading to the adoption of strategies typically utilized for multiple myeloma, which, however, exhibit limited efficacy. Selinexor is considered effective in treating relapsed/refractory multiple myeloma, but there are currently no reports on its application in primary plasma cell leukemia. Here, we reported a case of primary plasma cell leukemia with multiple high-risk genetic factors (including 1q21+, 17p‐, and 13q‐) who received a chemotherapy regimen including selinexor, pomalidomide, and dexamethasone.

**Patient concerns:**

This case was a 58-year-old male presenting with lower back pain, abdominal pain, and various systemic symptoms.

**Diagnoses:**

The initial diagnosis of intestinal obstruction at a local hospital was followed by a referral to our emergency department due to abnormal blood test results indicative of a hematologic disorder. Further investigations confirmed a rare diagnosis of primary plasma cell leukemia of the IgA-k light chain subtype.

**Interventions:**

The patient was promptly treated with a chemotherapy regimen comprising selinexor, pomalidomide, and dexamethasone in addition to supportive care.

**Outcomes:**

Subsequent assessments showed a significant response to treatment, with improvement in symptoms, normalization of blood parameters, and achievement of very good partial response. However, due to financial constraints, the patient declined hematopoietic stem cell transplantation and eventually opted to discontinue treatment, leading to disease progression.

**Lessons:**

The combination of selinexor with pomalidomide and dexamethasone has shown good efficacy in primary plasma cell leukemia with high-risk genetic abnormalities. Our case may provide evidence for developing an effective selinexor-based regimen for treating primary plasma cell leukemia with high-risk genetic abnormalities.

## 1. Introduction

Plasma cell leukemia (PCL) is a rare and highly aggressive plasma cell disorder, accounting for 0.5% to 4% of patients with multiple myeloma (MM).^[1]^ Based on the presence of MM history, PCL can be classified into primary PCL (pPCL) and secondary PCL. Currently, there is no recognized treatment protocol for PCL, and the MM treatment approaches are typically used. In the era of new drug therapies, although some proteasome inhibitors can improve the poor prognosis of PCL patients, the overall prognosis remains unfavorable.^[1]^ This could be related to the presence of high-risk genetic variations, which are more commonly detected in pPCL patients than in MM patients.^[2]^ In MM patients, 1q21+ is considered a high-risk genetic abnormality and is an independently poor prognostic factor. Many genes at the 1q21 locus can lead to early disease progression and resistance to MM therapy. Neither high-dose chemotherapy, the use of proteasome inhibitors, nor immunomodulators can overcome the adverse prognostic impact of 1q21+.^[3]^ However, selinexor, as the first novel oral selective inhibitor of nuclear export, has been shown to improve progression-free survival (PFS) in patients with 1q21+.^[4]^ Selinexor demonstrates good efficacy in relapsed/refractory MM, including those with high-risk cytogenetic abnormalities or resistance to multiple therapies, and shows synergistic effects when used in combination with other therapeutic drugs.^[5,6]^ However, there are currently no reports on its application in pPCL.

This study reports a patient with pPCL and multiple high-risk genetic factors (including 1q21+, 17p‐, and 13q‐) who received combination therapy of selinexor with pomalidomide and dexamethasone (SPD). After 1 treatment cycle, the patient achieved a very good partial remission, with improved clinical symptoms. After 3 treatment cycles, there was complete remission. This treatment regimen demonstrated unexpectedly rapid hematologic remission in this newly diagnosed pPCL patient complicated with multiple high-risk genetic abnormalities, providing a clinical reference for pPCL treatment.

## 2. Case presentation

**Figure 1.**
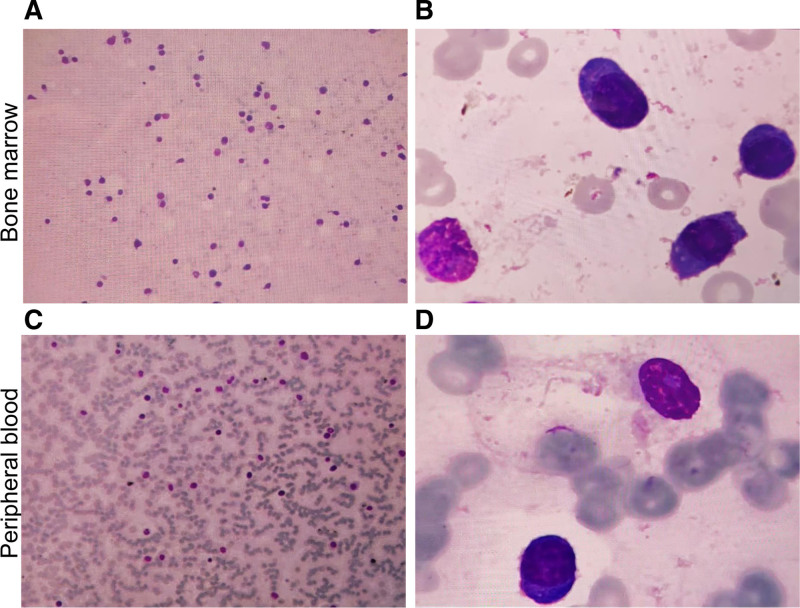
Bone marrow and peripheral blood smears results. (A) The primitive and immature plasma cells accounted for 25% of bone marrow, and mature plasma cells accounted for 46% (×10 magnification); (B) morphology of plasma cells in bone marrow: varying cell sizes, relatively regular nuclear shape, blurred nucleoli, and presence of binucleated plasma cells (×100 magnification); (C) peripheral blood smears showed that the primitive and immature plasma cells accounted for 21%, while mature plasma cells accounted for 50%; (D) morphology of peripheral blood plasma cells (×100 magnification).

**Figure 2.**
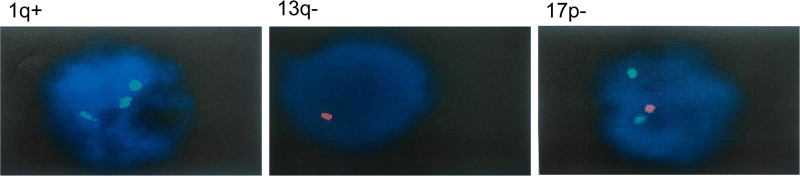
Bone marrow fluorescence in situ hybridization. The positivity rates for 1q21+, 13q‐, and 17p‐ were 82%, 78%, and 83%, respectively.

A 57-year-old male presented with lower back pain, abdominal pain, constipation, fever, chills, dizziness, and fatigue since July 2023. He was initially diagnosed with intestinal obstruction at a local hospital and received supportive treatment including fasting, bowel lubrication, intravenous nutrition, infection control, and maintenance of internal environment stability. Following symptom relief, he was discharged but continued to experience dizziness, fatigue, and gum bleeding. Subsequent blood tests at the local hospital revealed significantly elevated white blood cell count and extremely low platelet count, prompting referral to our emergency department. On admission, the blood routine tests showed a white blood cell count of 38.21 × 10^9^/L, hemoglobin of 65.00 g/L, and platelet count of 17.00 × 10^9^/L. Liver function tests indicated aspartate aminotransferase of 64.65 U/L, alanine aminotransferase of 116.00 U/L, and albumin of 28.90 g/L. The kidney function tests revealed urea of 13.00 mmol/L, creatinine of 273.80 μmol/L, and uric acid of 844.92 μmol/L. The electrolyte levels showed potassium of 3.36 mmol/L and calcium of 3.98 mmol/L. β2-microglobulin was 10.99 mg/L and lactate dehydrogenase was 1742.74 U/L. Immunofixation electrophoresis indicated IgA-κ type M proteinemia with an M protein content of 15.42 g/L. For serum-free light chains, there was κ chain of 216.09 mg/L, λ chain of 8.35 mg/L, and κ/λ ratio of 25.879. Bone marrow smear results (Fig. 1A and B) showed that primitive and immature plasma cells constituted 25% of the total, with varying cell sizes and relatively regular nuclear shapes. Blurry nucleoli and bi-nucleated plasma cells were observed. Mature plasma cells accounted for 46%. Peripheral blood smear results (Fig. 1C and D) revealed that primitive and immature plasma cells constituted 21%, while mature plasma cells accounted for 50%. Immunophenotyping analysis demonstrated that CD38st+ CD138st+ cells, making up 77.37% of nuclear-containing cells, expressed CD38st, CD138st, CD117part, CD33, CD13dim, CD20part, and cKappa, while lacking CD7, CD45, CD56, CD34, CD10, CD19, CD11b, CD4, CD8, CD3, CD22, CD14, CD64, and lambda. This suggests an abnormal phenotype of monoclonal plasma cells. Chromosomal examination indicated a karyotype of 46XY with dup(1)(q21q32) and del(13)(q12q22)[1]/46XY[1]. The fluorescence in situ hybridization revealed the positivity rates for 1q21+, 13q‐, and 17p‐ were 82%, 78%, and 83%, respectively (Fig. 2). Second-generation gene sequencing of bone marrow indicated a TP53 gene mutation frequency of 80.7%. Whole-body flat bone X-rays and full spine MRI showed no signs of osteolytic destruction. Thoracoabdominal pelvic CT revealed mild patchy infiltrates in both lungs, along with bilateral pleural and pericardial effusions. Additionally, there was subcutaneous fat space opacity around the chest wall, back, and pelvic region. Enlarged lymph nodes were observed in the mediastinum, bilateral axilla, retroperitoneum, mesentery, and bilateral inguinal regions, along with splenomegaly. Based on the patient’s medical history and laboratory examinations, the definitive diagnosis of pPCL of the IgA-k light chain subtype was made.

To rapidly reduce the tumor burden and alleviate the symptoms, the patient was given an SPD chemotherapy regimen involving the combination of selinexor, pomalidomide, and dexamethasone. The specific drugs and doses were as follows: selinexor 60 mg orally on days 1, 8, 15, and 22; pomalidomide 4 mg orally once daily (days 1–21, with a 1-week break); dexamethasone 40 mg orally once a week, with a 28-day cycle. Concurrent treatments included anti-infection therapy, correction of electrolyte imbalances, liver, and kidney protection, correction of hypoalbuminemia, parenteral nutrition, and blood component transfusions. After 1 treatment course, the patient’s symptoms improved, with blood tests indicating normal blood cell counts. Both bone marrow and peripheral blood smears did not reveal any primitive or immature blast cells. Immunofixation electrophoresis showed a minor amount of IgA-k type M proteinemia (M protein level of 1.3 g/L, a 91% decrease from previous levels). Minimal residual disease testing revealed that the level of abnormal cells was 1.29 * 10^–3^. The evaluation demonstrated a very good partial remission, prompting the continuation of the SPD regimen. Following 3 courses, a complete remission was confirmed upon reassessment. The hematopoietic stem cell transplantation was recommended for this patient. However, due to financial difficulties, the patient declined the procedure. The ongoing administration of the SPD regimen was then advised for disease management. Nevertheless, the patient independently discontinued selinexor. Subsequent readmission for a follow-up in November 2023 revealed disease progression. The patient refused further treatment and requested to be discharged.

## 3. Discussion

pPCL is the rarest and most aggressive disease in the spectrum of plasma cell disorders, often with features of MM and acute leukemia. It presents with more complex clinical manifestations and strong invasiveness, with more common extramedullary infiltration, the percentage of which may exceed 20%.^[7]^ However, while bone destruction is uncommon. Extramedullary infiltration can occur in various tissues such as liver, spleen, lymph nodes, serous cavities, soft tissues, central nervous system, etc, often accompanied by kidney failure, hypercalcemia, thrombocytopenia, elevated lactate dehydrogenase levels, and increased plasma cell labeling index.^[8]^ In this report, the patient clinically presented with pleural and pericardial effusions, lymphadenopathy, and splenomegaly. After treatment, the effusion was absorbed and there was a reduction in the size of the lymph nodes and spleen, suggesting a high possibility of extramedullary infiltration. However, there was a lack of pathological results to confirm this. The incidence of adverse genetic abnormalities in pPCL is higher than in MM, especially 13q, 17p deletions, 1q21+, and t(14;16) translocations, indicating that pPCL has biological features of high-risk MM.^[2]^ The risk of high-risk genetic abnormalities can be cumulative, and compared to patients with solely t(14;16), those with del(17p), del(13q), and 1q21+ have significantly increased risks of progression or death.^[9]^ The more high-risk genetic abnormalities present, the worse the prognosis for patients. An et al^[10]^ found that patients with 1q+ had a poorer prognosis and showed early progression during bortezomib treatment, suggesting that 1q+ may lead to resistance to bortezomib. A retrospective study by Abdallah et al^[11]^ confirmed that regardless of the number of copies of 1q, both PFS and overall survival decreased. Patients with 1q+ have poorer survival rates regardless of other cytogenetic abnormalities, and bortezomib and immune modulators cannot improve the prognosis of patients with 1q+.^[12]^ The patient we reported had the combination of 1q21+, 13q‐, and 17p‐ at initial diagnosis, indicating a poor prognosis. The bone marrow samples from the patient exhibited a TP53 gene mutation identified through high-throughput sequencing technology. The loss of the TP53 gene, which accompanies the loss of 17p, disrupts the G1/S phase checkpoint function and plays a critical role in promoting genomic instability in pPCL.^[13]^ Nalghranyan et al^[14]^ demonstrated that whole exome sequencing of CD138+ cells isolated from PCL patients revealed that 75% of plasma cells with TP53 copy deletion could manifest TP53 mutation. This biallelic inactivation of TP53, termed “double-hit,” is associated with a dismal prognosis despite modern therapies. It significantly reduces both PFS and overall survival in pPCL patients.^[13,15]^ Therefore, a combination regimen incorporating new drugs should be contemplated to enhance the prognosis in patients with the double-hit TP53 mutation.

Selinexor, as an oral formulation, has high patient compliance. Early clinical studies^[5,6,16]^ have shown that selinexor has good efficacy in patients who have previously received lenalidomide, proteasome inhibitors, and those with high-risk cytogenetics (including del[17p] or 1q21+), significantly improving patients’ prognosis. The clinical benefits are more pronounced in patients in the early stages of the disease and those who have not received multiple-line therapies.^[16]^ The patient in our report was diagnosed with pPCL of IgA-k light chain type. Fluorescence in situ hybridization analysis revealed 1q21+, 13q‐, and 17p‐, indicating multiple high-risk genetic factors and a poor prognosis for the patient. To rapidly reduce the tumor burden, we promptly initiated a novel combination therapy based on selinexor. As expected, this newly diagnosed pPCL patient showed rapid hematologic responses after treatment with the SPD regimen but relapsed during consolidation therapy, possibly associated with multiple high-risk genetic abnormalities. The rapid and lasting response for patients with multiple high-risk genetic abnormalities remains a key issue that urgently needs to be addressed. The FORTE study^[17]^ found that there was a longer PFS for 1q+ patients who underwent sequential autologous stem cell transplantation (ASCT) compared to those receiving chemotherapy alone, indicating that ASCT can improve the survival of 1q+ patients. For 1q+ patients eligible for transplantation, a regimen based on selinexor combined with ASCT is recommended to prolong survival and improve the quality of life.

## 4. Conclusion

In conclusion, this case report demonstrates the successful use of a novel combination therapy involving selinexor, pomalidomide, and dexamethasone in a patient with pPCL and multiple high-risk genetic factors, including 1q21+, 13q‐, and 17p‐. The treatment regimen led to rapid hematologic responses, including a very good partial remission after 1 cycle and complete remission following 3 cycles, highlighting the potential of personalized approaches in addressing the adverse effects of high-risk genetic variations in pPCL. The case emphasizes the need for further research on individualized therapeutic interventions, such as combining selinexor with ASCT, to enhance outcomes for patients with pPCL and high-risk genetic profiles.

## Author contributions

**Conceptualization:** Wenxia Fan, Xinhong Guo.

**Data curation:** Wenxia Fan, Ying Liu, Ming Jiang.

**Funding acquisition:** Xinhong Guo.

**Investigation:** Wenxia Fan, Lei Wang, Xinyou Wang, Ying Liu, Mingling Sun, Nadia Abduklimu, Rui Zhang, Ming Jiang.

**Methodology:** Wenxia Fan.

**Project administration:** Xinhong Guo.

**Resources:** Wenxia Fan, Lei Wang, Xinyou Wang, Mingling Sun, Nadia Abduklimu, Rui Zhang, Xinhong Guo.

**Supervision:** Xinhong Guo.

**Writing – original draft:** Wenxia Fan.

**Writing – review & editing:** Lei Wang, Xinyou Wang, Ying Liu, Mingling Sun, Rui Zhang, Ming Jiang, Xinhong Guo.
